# Nutrition Program Fidelity Assessment tool: a framework for optimising implementation in military dining facilities

**DOI:** 10.1017/S1368980022001896

**Published:** 2022-10-13

**Authors:** Katie M Kirkpatrick, Carolyn A Kleinberger, Elizabeth M Moylan, Asma S Bukhari, Patricia A Deuster

**Affiliations:** 1 Consortium for Health and Military Performance, Department of Military and Emergency Medicine, F. Edward Hébert School of Medicine, Uniformed Services University, Bethesda, MD, USA; 2 Henry M. Jackson Foundation for the Advancement of Military Medicine, 6720B Rockledge Drive, Suite 605, Rockville, MD 20817, USA; 3 Military Nutrition Division of the US Army Research Institute of Environmental Medicine, Natick, MA, USA

**Keywords:** Dining facility programmes, Nutrition implementation science, Programme assessment tools, Traffic light labelling

## Abstract

**Objective:**

The aim was to develop, refine and assess the usefulness of the Go for Green^®^ (G4G) 2.0 Program Fidelity Assessment (PFA) tool. G4G 2.0 is a Department of Defense programme designed to optimise access, availability and knowledge of high-performance nutritious foods in military dining facilities (DFAC).

**Design:**

During a multi-site study to evaluate G4G 2.0 on meal quality and diner satisfaction, subject matter experts developed and refined a PFA tool based on eight programme requirements (PR). They identified tasks critical to programme success and corresponding benchmarks, then proposed expansion of several PR and developed a scoring system to assess adherence. Three PFA were conducted (Site 1, Site 2A and Site B).

**Setting:**

Two DFAC in the USA implementing the G4G 2.0 programme.

**Participants:**

Military DFAC participating in a G4G 2.0 evaluation study.

**Results:**

After G4G 2.0 implementation, Site 1 conducted a PFA and met benchmarks for eight of fifteen sections. At Site 2, a PFA was conducted after G4G 2.0 implementation (Site 2A) and one 3 months later (Site 2B) with twelve of fifteen and ten of fifteen sections meeting benchmarks, respectively.

**Conclusion:**

Research highlights the need to maximise implementation quality to ensure interventions are effective, achievable and efficient. Using a PFA tool to objectively assess nutrition interventions can inform programme fidelity, successes and opportunities for improvement. Results identify key areas that require additional training and resources to optimise access to nutrient-dense foods that support nutritional fitness. This feedback is critical for assessing potential programme impact on Service Members.

The effectiveness of nutrition interventions using point of service labelling and food choice architecture is well-established^(1–7)^. Several studies show positive changes in diners’ food choices and health-related behaviours^(8–10)^. Department of Defense research has shown that many Service Members do not meet the standards for consuming the nutrient-dense, high-performance foods necessary to optimise health and mission readiness^(11–13)^. The Department of Defense developed a comprehensive nutrition programme, Go for Green^®^, to improve the nutritional fitness of Service Members consuming meals at military dining facilities (known as DFAC)^(14–18)^. The programme consists of menus with traffic light colour labels indicating performance impact, choice architecture to encourage easy access to high-performance food choices and education to promote these choices. The current programme, Go for Green^®^ version 2.0 (G4G 2.0), has been iteratively revised and rebranded from the original G4G, which consisted of food labelling and posters only^(19)^. Most notably in 2017, the following programme requirements (PR) were added to the original ones: an updated validated, standardised coding algorithm for trained users, initial and ongoing food service staff training and comprehensive marketing strategies for a total of eight PR. The PR were reviewed and approved by each military branch foodservice headquarters before utilisation in programme quality assessment. Nutrition interventions such as G4G can be useful tools to provide a supportive food environment where Service Members live, work and train.

After conducting a 2016 G4G programme evaluation as part of US Army Performance Triad pilot sites, the Army Public Health Center team made a recommendation to ‘create benchmarks that are measurable objectives for programme implementation requirements^(20)^’. This prompted development of a comprehensive tool to assess quality and compliance with PR, identify gaps and account for inconsistencies among different DFAC. Research supports the use of a tailored evaluation tool to strengthen the quality and impact of implementation and ensure intervention goals are achievable^(21)^. To that end, the G4G Program Office at the Consortium for Health and Military Performance, Uniformed Services University (providing nutrition and military DFAC expertise) developed the first version of the G4G 2.0 Program Fidelity Assessment (PFA) tool in collaboration with the Army Public Health Center (providing programme evaluation expertise). The PFA is the first evaluative tool in G4G’s 10-year history.

Across 2017–2019, G4G 2.0 underwent formal evaluation at two military DFAC to assess effectiveness in improving meal quality without compromising diner satisfaction^(19)^. The G4G Program Office assisted DFAC with G4G 2.0 planning and implementation; this provided an opportunity to pilot the newly developed PFA and make iterative refinements (Fig. 1). During site visits by the G4G Program Office, hands-on assistance to DFAC food service staff and management, in addition to remote support, was identified as critical to programme implementation and sustainment. The PFA served as a tool to evaluate how well each DFAC executed G4G 2.0 according to the established PR at the following time points: Site 1 (September 2018), Site 2A (June 2019) and Site 2B (September 2019).

**Fig. 1:**
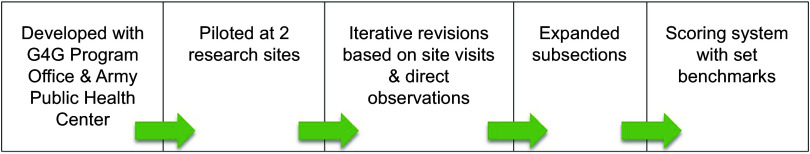
G4G 2·0 Program Fidelity Assessment refinement process. G4G, Go for Green^®^

This article describes the iterative PFA development and the results of piloting this tool during G4G 2.0 implementation at two military DFAC. Foodservice operation constraints are highlighted along with recommended strategies to enhance G4G 2.0 programme implementation across the Department of Defense.

## Methods

### Program Fidelity Assessment instrument description and refinement

The G4G 2.0 PFA outlines eight PR, approved by the military food operations leadership, to enhance programme consistency and standardisation (hprc-online.org/nutrition/go-green/g4g-getting-started/implementation/program-requirements/program-requirements-pdf). The eight PR are (1) standardised training for management, (2) assign traffic light colour codes, (3) G4G 2.0 menu targets: minimum Green-coded items, (4) standardised food cards, (5) food-placement strategies, (6) promotion of Green-coded foods, (7) marketing and education and (8) standardised training for all staff.

First, the G4G Program Office identified actionable tasks that aligned with the above stated eight PR to evaluate whether the PR was met. Each PR task provides an objective measure of compliance through verification methods (direct observation or retrospective review of food production and service documents). The PFA was then refined during early G4G 2.0 implementation visits to improve tool usability (formatting, consistent language) with minimal content revisions. The PFA tool validity was established through involvement of subject matter experts in this topic area. Also, user instructions were revised to improve the reliability of the responses as intended. The initial scoring system involved ‘yes’ and ‘no’ checkboxes for each PR.

During G4G 2.0 implementation, evaluators collected a large amount of data from each DFAC research site that was not captured on the original PFA at Site 1. Given the value of the information collected, the G4G Program Office identified gaps in the PFA which facilitated the creation of several detailed subsections. In response, the PFA subsections were expanded to more accurately assess implementation and identify critical tasks that could contribute to programme success (Table 1). For example, PR3 states a minimum of at least one Green-coded item for each meal component must be offered at every station. Although the DFAC menu appeared to meet this target, over the course of the study, this was determined to be inaccurate. The actual recipes were not followed (staff used altered recipes or changed food preparation practices), which sometimes yielded a ‘lower’ colour code than originally intended. Menu items were also replaced with less nutritious substitutions. Therefore, the served menu offered less high-performance foods than the benchmark. As a result, new task items were added to PR3 to better assess menu and recipe fidelity. Additionally, gaps were identified in G4G 2.0 programme marketing and education (PR7) and staff training (PR8) (Table 1).

**Table 1:** Program Fidelity Assessment (PFA) tool expanded sections

Programme requirement	Original PFA item	Gap identified based on direct observations	Final PFA items
PR3 – menu targets: minimum Green-coded items	30 % Green-coded menu		3·1: Minimum Green-coded items
		Planned menu is not always served menu	3·2: Planned *v*. served menu
		Recipes not being followed	3·3: Recipe fidelity
PR7 – marketing and education	Printed material (posters, table tents/signs, brochures)		7·1: Print materials
		Missed marketing and education opportunities	7·2: Social media
			7·3: G4G 2.0 grand opening
			7·4: Post-grand opening
			7·5: Nutrition education
PR8 – standardised training for all staff	80 % of staff trained on G4G		8·1: Led by certified staff trainer
		Not capturing enough detail, not encouraging different strategies to optimise training	8·2: Standardised slide deck training
			8·3: Hands-on training

PR, programme requirement; G4G, Go for Green^®^.

### Testing the Program Fidelity Assessment at two locations

At the two sites, the PFA was conducted by the G4G Program Office and the installation registered dietitians to align with specific research time points. The evaluator was trained on how to complete the PFA to ensure an understanding of the scoring system.

The PFA requires direct observation of the kitchen (recipe creation and meal prep) and DFAC meal service operations (food availability and set-up at each serving station, such as mainline and salad bar) over a minimum of two meal periods. Additionally, evaluators required DFAC staff assistance in between meal service to access menus, training plans and records, and marketing efforts. During their review, evaluators noted the number of correct actions and awarded one point each.

### Scoring the Program Fidelity Assessment: comparing observations to benchmarks

The next major revision included expansion of the scoring system to objectively assess PR fidelity. Information gleaned from the concurrent research evaluation about programme implementation further informed the PFA scoring system. The G4G Program Office determined realistic and achievable benchmarks for the military setting for each PR section based on programme goals along with extensive knowledge of military food service operations. Benchmarks critical to programme integrity were set at 100 %. Other benchmarks for which less than 100 % would be acceptable to programme quality were set at 75 % or 80 %, to account for the realistic variability in daily programme execution. Table 2 provides a detailed breakdown of the major tasks and PR benchmarks. A full-length version of the PFA is available upon request.

**Table 2:**
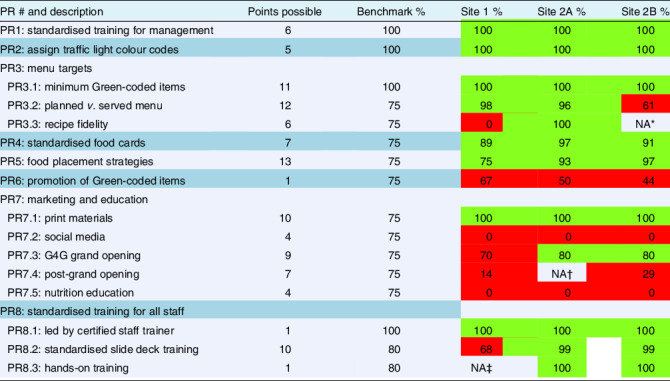
G4G 2.0 programme requirements with benchmarks and results from two sites

PR, programme requirement.

*NA = data not collected.

†NA = not applicable due to review time period.

‡NA = not provided to site.

Scored sections = Green or red highlight; green highlight = PR compliance at or above benchmark; red highlight = PR compliance below benchmark.

Site 1 and Site 2A: PFA conducted immediately after G4G 2.0 implementation; Site 2B: PFA conducted 3 months after G4G implementation.

#### PR1: standardised training for management

The PFA tool captures the G4G 2.0 related training. The requirement is for local G4G team leads to complete the G4G 2.0 PR and G4G 2.0 staff trainer online training courses. The staff assigned to menu modifications and colour coding are required to complete the G4G 2.0 coder training. The evaluators conducted a review of training certificates to confirm completion. Well-trained team leads are vital to successful G4G 2.0 implementation; therefore, the benchmark was set at 100 %.

#### PR2: assign traffic light colour codes

The DFAC menu (mainline, specialty bars) was reviewed to ensure a certified G4G 2.0 coder assigned all foods with both colour and Na codes (beverages and fruits were assigned colour codes only). Accurate coding ensures programme integrity and builds consumer trust; therefore, the benchmark is 100 %.

#### PR3: menu targets: minimum Green-coded items

**Fig. 2:**
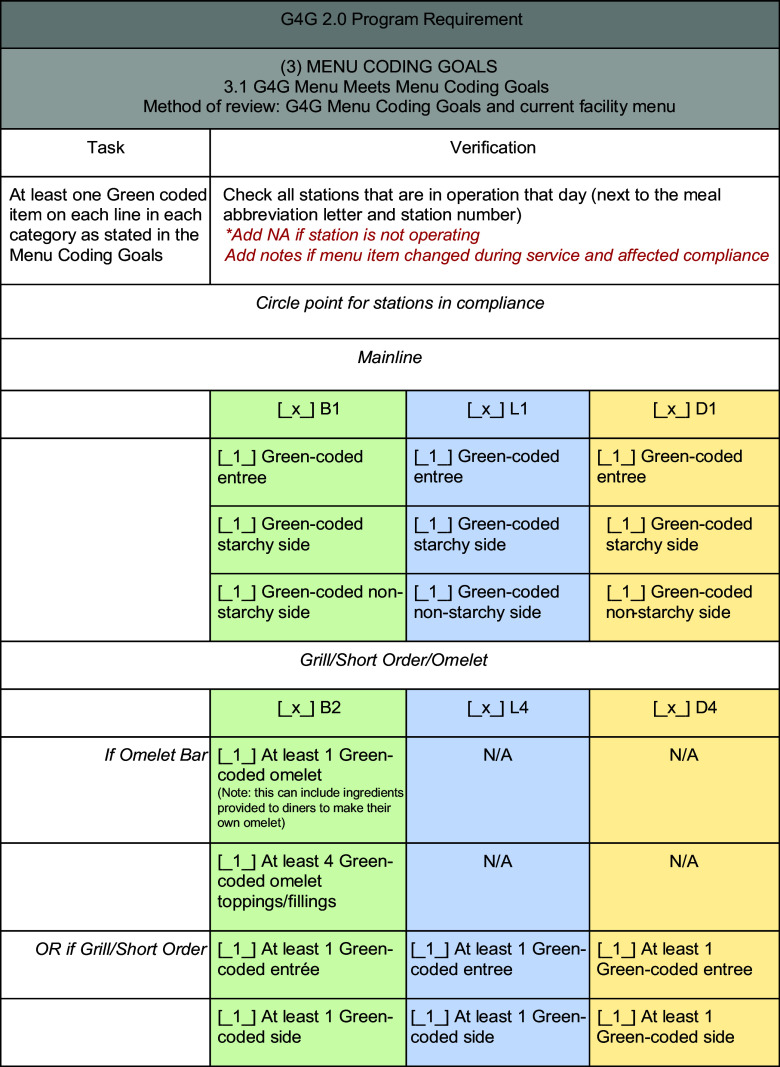
Subsection of the Program Fidelity Assessment to assess number of Green-coded items offered at serving stations. G4G, Go for Green^®^; B, breakfast serving station option; L, lunch serving station option; D, dinner serving station option

Evaluators conducted an in-depth review of the G4G 2.0 menu to confirm the presence of at least one Green-coded food within each meal component: entree, starchy vegetable and non-starchy vegetable on each serving station. Other stations were assessed by percentage Green: salad bar (50 %), deli, dessert and beverage stations (30 %). The PFA provides a step-by-step breakdown to check the menu against the benchmark of 100 % (Fig. 2).

The evaluator compared the planned menu, as written, to the served menu, as observed, in real time. Every planned item should match the served item or be replaced with an appropriate substitution, a menu item of equal or ‘better’ colour code. For example, a Green-coded starch, brown rice, could be substituted with another Green-coded starch (roasted potato wedges) but not with Yellow-coded white rice. Missing items and inappropriate substitutions (items of ‘lesser’ colour code) were counted against the score. An itemised list of menu items and served items were recorded in the PFA to assess against the 75 % benchmark (Fig. 2).

An audit of five recipes over two meal periods assessed the extent to which the recipe was prepared against the benchmark of 75 %. The audit confirmed the source where the cooks obtained the recipe (the military information management system, generic online recipe, from memory, etc.), how they prepared the item and what substitutions were used, if any. Deviations from the recipe card might alter the menu item’s colour and/or Na code and should be avoided to maintain accuracy and reliability of G4G 2.0 codes. Some substitutions are appropriate, including replacing ingredients, when necessary (if the same colour code) or the addition of seasonings (fresh herbs, citrus juice), as desired.

#### PR4: standardised food cards

Evaluators conducted a walk-through of serving areas to directly observe food card placement and compare against the 75 % benchmark. Food cards are the point-of-selection indicators for menu item name, performance impact (Green = high performance, Yellow = moderate performance and Red = low performance) and Na content (low, moderate and high). The evaluator confirmed if food cards were neatly displayed, available for every menu item and matched the menu items (‘pancake’ card in front of pancakes).

#### PR5: food placement strategies

Meal period observation identified if three total food placement strategies were implemented at least 75 % of the time. First, Green-coded items should be offered first, then Yellow and lastly Red. Alterations are permitted to assist with service flow. As an example, for meals requiring a base item (pasta or tortilla), a Yellow-coded base item (white pasta) can be placed before Green-coded toppings (roasted vegetables). Provided menus included visual menu ‘planograms’ to illustrate the optimal food placement per serving station.

Two additional food-placement strategies and/or menu revisions to highlight Green-coded menu items were assessed for each serving line. Examples include (1) offering two non-starchy vegetables; (2) offering wholegrain versions of refined grains and placing them first in line; (3) placing white bread out of sight; (4) offering grilled chicken, in addition to Yellow or Red-coded proteins, on the grill; (5) placing Red-coded items in smaller containers or out of sight and (6) offering infused water.

#### PR6: promotion of Green-coded items

**Fig. 3:**
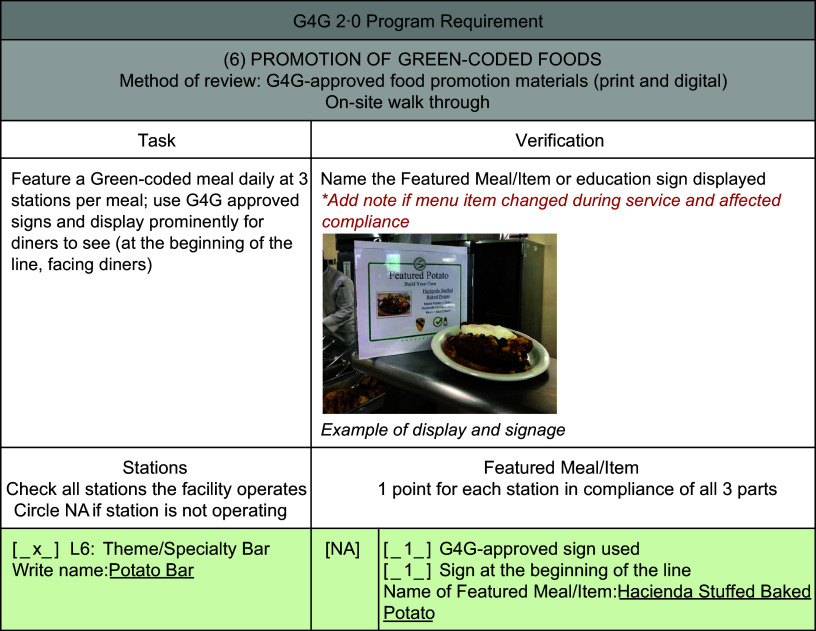
Subsection of programme fidelity tool to assess promotion of Green-coded foods. G4G, Go for Green^®^; L, lunch serving station option

The evaluator examined if a featured meal and corresponding sign were displayed at the beginning of the line or other prominent location for diner review prior to selection (Fig. 3). Three featured meals and signage should be present at every meal period (breakfast, lunch and dinner) and match the available items at least 75 % of the time. Featured meal examples include infused water, specialty bar dishes (salad bar, deli, theme bars) and Green-coded mainline meal (breakfast, lunch, dinner hot meal). Sample plates are encouraged, but not required, to increase the visual appeal.

#### PR7: marketing and education

Five marketing and education subsections were evaluated against a 75 % benchmark. Upon DFAC walk-through, PR7.1 examined if professionally printed, standard G4G 2.0 printed materials (posters, table tents/signs, napkin holders, brochures) were posted or available. At least three permanent posters should be displayed along with table tents or napkin holders neatly on tables, and brochures conveniently available. Additionally, rotating posters regularly add fresh content. An audit of the facility’s social media account determined if PR7.2 met posted content at least twice per week. This section was ‘not applicable’ if the DFAC could not post to a social media account.

The PR7.3 subsection addressed the G4G 2.0 marketing plan, including events and activities for the grand opening day with promotions inside the DFAC and installation wide. News articles or press releases, social media, printed materials, flyers and emails are important marketing strategies. Due to the military setting, marketing strategies should involve the public affairs office for maximum exposure and for regulatory adherence. PR7.4 assessed the G4G 2.0 marketing plan sustainment after the grand opening.

PR7.5 assessed whether the installation registered dietitian or other nutrition professional presented nutrition and G4G 2.0 education to the supported military units with date, method, number of attendees, instructor and lesson plan details recorded. DFAC are encouraged to report programme implementation and menu changes to local registered dietitians and unit-level leadership for situational awareness and synergy with other training, initiatives and campaigns.

#### PR8: standardised training for all staff

A detailed count of training records against the DFAC staff roster was conducted. The G4G 2.0 staff trainer provided the training sign-in rosters along with topics or training modules covered and time spent. Because of high DFAC staff turnover, regularly scheduled training sessions are recommended to capture new staff and refresh current staff to achieve the 80 % benchmark.

### Data analysis

Data analysis included comparisons of achieved scores against set benchmarks for each PR at one time point for Site 1 and two time points for Site 2 (2A and 2B). Following completion of each section, all scores were totalled and the final percentage compared against the benchmark. Results were denoted as either compliant (at or above the established benchmark) or non-compliant (below the established benchmark for each PR).

## Results

The G4G Program Office collaborated with DFAC staff and management at two military study sites to execute G4G 2.0 planning, implementation and sustainment. The implementation process resulted in the development and refinement of content and benchmarks to objectively assess programme fidelity by using the first G4G PFA tool. For accurate comparison, certain data from Site 1 were retroactively added to the expanded PFA version utilised at Site 2. During the study, the G4G Program Office conducted in-person management training (PR1), assigned colour and Na codes to a 21-d menu with twelve specialty bar options (PR2) to meet G4G 2.0 menu targets (PR3) and printed materials and other marketing strategies (PR7).

After a phased implementation, Site 1 PFA results indicated that the DFAC successfully met the benchmarks for eight of fifteen scored sections (Table 2). At Site 2, the PFA was conducted and scored separately at 2 time points: the grand opening (Site 2A) and again 3 months later (Site 2B). Although the time between the PFA was short, the initial (grand opening) PFA followed a period of close support and guidance from the G4G Program Office. Per the initial PFA, the DFAC successfully met the benchmarks for twelve of fifteen scored sections. The second PFA indicated the DFAC met benchmarks in ten of fifteen scored sections (Table 2). The detailed results for Site 1, Site 2A and Site 2B are discussed below.

### PR1: standardised training for management

Both sites received a 100 % score as in-person training was conducted by the G4G Program Office and online training accessed through an online learning management system (hprc-online.org/nutrition/go-green/g4g-getting-started/training-classes).

### PR2: assign traffic light colour codes

A G4G Program Office certified G4G 2.0 coder assigned colour and Na codes for all Armed Forces Recipe Service recipes utilised in the menus to meet the 100 % requirement.

### PR3: menu targets: minimum Green-coded items

The planned menu provided the target number of Green-coded items for both sites, resulting in a 100 % score for PR3.1. Staff served the menu as written and provided appropriate substitutions (PR3.2) at Site 1; however, the actual recipes used were not the correct updated recipes from the provided menu (PR3.3). The outdated recipes did not reflect colour or Na codes of updated recipes so, although the planned menu met the required standards, the served menu did not. The average score for three subsections resulted in a PR3 total score of 66 %.

At Site 2, the planned *v*. served menus (PR3.2) were inconsistent. The grand opening PFA (Site 2A) indicated a recipe fidelity of 96 % as the menu was served as planned with acceptable substitutions. The post-grand opening PFA (Site 2B) showed the recipe fidelity dropped to 61 % due to missing items and inappropriate substitutions (‘lesser’ colour code) on the serving line. Observation of eight recipes on the serving station during the grand opening provided a 100 % score for recipe fidelity (PR3.3); recipe data were not captured in the Site 2B PFA. Averaging the subsections together, the PR3 total score was 99 % for Site 2A and 81 % for Site 2B.

### PR4: standardised food cards

Site 1 received an 89 % for displaying neat and organised, available and accurate food cards, whereas Site 2 received a 97 and 91 % at the first and second time points, respectively.

### PR5: food placement strategies

Site 1 was successful in setting up food items in the recommended order (Green, Yellow, Red) at the mainline but inconsistent at specialty bars. In fact, only seven of nineteen were in the correct order. In contrast, during Site 2 time points all the serving lines including specialty bars were set up correctly. Upon evaluation, both sites successfully utilised two additional strategies to nudge diners towards nutritious options. The scores were 75 % (Site 1), 93 % (Site 2A) and 97 % (Site 2B).

### PR6: promotion of Green-coded items

Featured meal signs that aligned with the 21-d rotating mainline menu and specialty bars were provided to both facilities. However, Site 1 scored 67 %, Site 2A scored 50 % and Site 2B scored 44 % in offering featured meals and posting signage.

### PR7: marketing and education

Site 1 was successful in posting printed materials (PR7.1) but unsuccessful with using social media (PR7.2) for promotion. A grand opening event was held with some promotional materials (email flyer, G4G 2.0 video), but without a clear marketing plan or utilising social media platforms many opportunities were missed. Also, G4G 2.0 marketing post-grand opening (PR7.4) was limited and no nutrition education (PR 7.5) was provided. The average score for all five subsections resulted in a PR7 total score of 37 %.

Site 2 was successful with implementing print materials and promoting the grand opening through the DFAC and public affairs office. However, social media was not used and DFAC, menu and G4G programme marketing did not continue after the grand opening. Lastly, nutrition education was not provided to supported units. At time point 1 (Site 2A), the average score for all five subsections resulted in a PR7 total score of 45 %. Similarly, at time point 2 (Site 2B), the total score was 42 %.

### PR8: standardised training for all staff

Training sessions were held 6 times over 10 months at Site 1, taught by the DFAC’s certified training lead or the G4G Program Office (PR 8.1), using the standardised G4G 2.0 training modules (PR8.2). Hands-on training was unavailable and thus not scored (PR8.3). Calculating trained *v*. available staff per rosters showed 68 % of staff were trained. The average score for three subsections resulted in a PR8 total score of 84 % for Site 1.

At Site 2, training sessions were held eight times over 7 months using the standardised modules along with small group hands-on training. Calculating against the staff roster, 99 % of Site 2 staff were trained at each PFA time point. The average score for all three subsections resulted in a PR8 total score of 99 % at Site 2 for both time points (2A, 2B).

## Discussion and conclusion

Implementation of any nutrition intervention, such as G4G 2.0, encounters challenges, particularly when trying to achieve full programme fidelity^(14,22,23)^ (Bukhari AS, unpublished results). Knowing the degree of programme fidelity is essential to understanding interventional impact, improving the programme and then extrapolating to other facilities. However, review of the current literature shows that fidelity/integrity assessments are not common. Metcalfe *et al.* reviewed twenty-nine school food intervention studies and found that only two (7 %) used strong intervention integrity checks, which consisted of unannounced researcher observations^(22)^. The majority (21/29 or 74 %) did not use any fidelity monitoring. This is clearly a gap in nutrition intervention implementation. Interestingly, programme fidelity is an issue in many health programmes. Schaap *et al*. reviewed programme fidelity of school-based obesity prevention programmes and found substantial variability in how fidelity was defined, how it was quantified and the overall scores (0–86 %), although most scores were low^(23)^. Methods included observations, logbooks and questionnaires. A 2020 review of ‘school meal nudge interventions’ indicated that methods incorporating implementation metrics are essential to address system factors and isolate individual intervention tasks for assessment^(22)^. The G4G 2.0 PFA was developed using individual intervention tasks to determine fidelity of established PR and identify barriers and facilitators for success.

Throughout the study period, specific PR (#3, 7, 8) were identified as notably challenging and it was decided to expand these PFA sections. For example, PR3 initially only captured fidelity of the minimum number of Green-coded menu item targets. The need for additional critical tasks arose from Site 1 observations that menu items served at the serving station did not align with the planned menu. Through further investigation, it was discovered staff served non-planned items for legitimate reasons (desire to use leftovers, running out of items, ingredients unavailable from the vendor) but sometimes made suboptimal substitutions. Observed challenges included non-adherence to recipes because the correct, updated G4G 2.0 recipes were not available in the information management system, nutritious ingredients were unavailable from vendors (Greek yogurt, zucchini, wholegrain pasta) or staff used their own preference or creative touches. This challenge was mitigated at Site 2 by successfully adding ingredients to the vendor catalogue and inputting the updated G4G 2.0 recipes into the information management system, which resulted in higher fidelity for this PR compared to Site 1. Unavailable ingredients and recipe adherence remain challenges.

Additionally, marketing DFAC and their enhanced menus to the larger installation audience (PR 7) was challenging, as these are not typically high priorities among foodservice leadership and would require installation support, although interest has been increasing. Likewise, setting aside time for DFAC staff training (PR8) is not easy, given the busy tempo of operations and focus on food production. Foodservice staff training is key to successful implementation of the enhanced menus and food placement strategies. The training reinforces the ‘why’ behind G4G 2.0 and improves staff buy-in to support and sustain the programme.

Considering these common challenges and potential solutions are important. The G4G Program Office shared the results with local G4G 2.0 and leadership teams to highlight successes and identify opportunities for improvement. The close involvement of the G4G 2.0 Program Office allowed for on-site assistance and provision of timely tailored resources and training to support low-scoring PFA areas. For example, at Site 2, three culinary skills training sessions were provided to teach or refresh basic cooking skills and learn and practise techniques utilised in updated recipes after culinary skills were identified as a gap at Site 1. The G4G 2.0 training modules, pre-coded recipes, pre-coded menus that meet menu targets and social media content are available via an open-access website (hprc-online.org/nutrition/go-green). These resources help facilitate G4G 2.0 implementation at installations worldwide, reducing required time, personnel and expertise locally.

Piloting the PFA identified programme implementation challenges and offered mitigation efforts to support a higher adherence to fidelity. For example, frequent recipe substitutions affect programme compliance. This was addressed by adding content to education and training on how to make appropriate recipe substitutions and the rationale behind the requirements (adherence to a performance-focused menu). The need for additional creative and innovative strategies for marketing, promotion, education and staff training was also identified. Using social media to communicate is one of the most popular ways Service Members receive, share and access information and should be further explored^(24)^. Encouraging communication about DFAC improvements (updated menus, new serving stations, food promotion, etc.) with Service Member and installation leadership can build programme trust and increase support and patronage to use DFAC as avenues to promote nutritional fitness. The areas scoring poorly on PFA highlighted underlying systemic challenges and will require engagement at a strategic level to ensure such programmes have the necessary support infrastructure.

Sarma *et al.* published ‘A conceptual framework for implementation science to evaluate a nutrition intervention scaled-up in a real-world setting’, which was a goal of the G4G Program Office^(25)^. The authors present a framework with five components: ‘1) identifying an “effective” intervention; 2) scaling-up and implementation fidelity; 3) course corrections during implementation; 4) promoting sustainability of interventions and 5) consideration of a comprehensive methodological paradigm to identify “effective” interventions and to assess the process and outcome indicators of implementation^(25)^’. The G4G 2.0 programme was examined with a research methodology resulting in positive outcomes. The next step requires an objective assessment of programme adherence with this newly validated PFA tool across more facilities.

Limitations exist for both the study methodology and PFA tool. Data were obtained from only two sites, both of which had close support and hands-on assistance from G4G Program Office staff, which is not realistic for all military DFAC implementing G4G 2.0. Additionally, direct observation and involvement from the G4G Program Office might impact the natural behaviour or usual performance of DFAC staff. Given the PFA development was iterative, some data at Site 1 had to be added and scored retroactively. The major limitations of the current PFA tool are time intensive, labour intensive and requiring skilled person(s) knowledgeable about the G4G 2.0 programme to conduct. Given this, the PFA tool requires a trained evaluator. Lastly, although the PFA does capture DFAC compliance with the G4G 2.0 programme at the time of assessment it does not either ensure or reflect long-term compliance. Several or regular assessments may need to be conducted to assess long-term programme adherence.

The G4G 2.0 PFA tool provides an objective measure of military DFAC performance in executing G4G 2.0. This tool was instrumental in identifying G4G 2.0 programme implementation challenges and gaps in the PFA tool itself and supported both G4G 2.0 programmatic and PFA tool improvements both during and post study. Beyond the Department of Defense, the presented PFA could be generalised and customised for use at similar institutional food settings (schools, universities, worksite cafeterias) as an effective public health tool to assess nutrition interventions designed to promote easily accessible, high-quality, nutrient-dense foods. The PFA is evolving with future research and refinement as needed to align with the ongoing revisions to the G4G 2.0 programme. Future studies should evaluate if all PR are equally necessary for achieving the desired outcomes. Strategies to enhance PFA usability through leveraging technology will facilitate immediate results needed to improve programme adherence and allow for larger applications. As the programme is scaled up across military installations, a PFA database will enable addressing challenges that impact foodservice operations. A comprehensive programme such as G4G 2.0 will require solid system-wide infrastructure to achieve and sustain the desired nutrition environment for the military community.
